# Phytochemical Screening and Polyphenolic Antioxidant Activity of Aqueous Crude Leaf Extract of *Helichrysum pedunculatum*

**DOI:** 10.3390/ijms10114990

**Published:** 2009-11-13

**Authors:** Olayinka A. Aiyegoro, Anthony I. Okoh

**Affiliations:** Applied and Environmental Microbiology Research Group (AEMREG), Department of Biochemistry and Microbiology, University of Fort Hare, Private Bag X1314, Alice 5700, South Africa; E-Mail: oaiyegoro@ufh.ac.za (O.A.A.)

**Keywords:** Helichrysum pedunculatum, scavenging, free radicals, antioxidant activity

## Abstract

We evaluated the *in vitro* antioxidant property and phytochemical constituents of the aqueous crude leaf extract of *Helichrysum pedunculatum.* The scavenging activity on superoxide anions, DPPH, H_2_O_2_, NO and ABTS; and the reducing power were determined, as well as the flavonoid, proanthocyanidin and phenolic contents of the extract. The extract exhibited scavenging activity towards all radicals tested due to the presence of relatively high total phenol and flavonoids contents. Our findings suggest that *H. pedunculatum* is endowed with antioxidant phytochemicals and could serve as a base for future drugs.

## 1. Introduction

Free radicals are chemically unstable atoms or molecules that can cause extensive damage to cells as a result of imbalance between the generation of reactive oxygen species (ROS) and the antioxidant enzymes [1]. Molecular oxygen is an essential component for all living organisms, where it helps in the process of oxidation which is a basic component of aerobic life and of our metabolism. Thus radicals are produced either naturally or by some biological dysfunction [2]. ROS or reactive nitrogen species (RNS) and their excess have a harmful effect, such as the peroxidation of the membrane lipids, aggression to tissue proteins and membranes, on damage to DNA and enzymes [1]. Therefore, they can be related to some pathologies, such as arthritis, hemorrhagic shock and coronary diseases, cataract, cancer and AIDS as well as age-related degenerative brain disorders [2]. The beneficial effects of antioxidants on promoting health is believed to be achieved through several possible mechanisms, such as direct reaction with and quenching free radicals, chelation of transition metals, reducition of peroxides, and stimulation of the antioxidative enzyme defense system [2].

Currently, there is a great interest in the study of antioxidant substances mainly due to the findings concerning the effects of free radicals’ in the organism. Phenolic plant compounds have attracted considerable attention for being the main sources of antioxidant activity, in spite of not being the only ones. The antioxidant activity of phenolics is mainly due to their redox properties, which allow them to act as reducing agents, hydrogen donors, and singlet oxygen quenchers. In addition, they have a metal chelation potential. The antioxidant activities of phenolics play an important role in the adsorption or neutralization of free radicals [3].

Several synthetic antioxidants are commercially accessible but have been reported to be toxic [4]. Plants have been reported to exhibit antioxidant activity due to the presence of antioxidant compounds such as phenolics, proanthocyanidins and flavonoids [5].

Different compounds like phenolics e.g. flavonoids and chalcones, phthalides, α-pyrone derivatives, terpenoids, essential oils, volatiles and fatty acids have been found in the genus *Helichrysum* [7] and biological activities of extracts from *Helichrysum* species have been widely reported [8–12]. However the compounds responsible for these activities have been identified in only a few cases. The scope of natural products that have been isolated from the *Helichrysum* genus is quite broad, covering nearly all the known fundamental classes, with the exception of alkaloids [11–13].

*Helichrysum* species, known as “impepho” in “isiXhosa”, and “everlastings” in English belong to the families Asteraceae and Compositae, and the genus consists of about 500 species, with 246 growing in South Africa [13]. The species are used according to their availability by geographical area. Prior to this study, there is no report on the antioxidant activity of *Helichrysum pedunculatum* in the available literature. This present study, therefore investigated the phytochemical compositions, the *in vitro* antioxidant and free radical scavenging potential of this plant.

## 2. Results and Discussion

### 2.1. Results

Investigation of the aqueous leaf extract of *H. pedunculatum* revealed the presence of tannins, flavonoids, steroids and saponins (Table 1). The total phenolic content of the aqueous leaf extract was 0.512 mg gallic acid equivalent/g of extract. The total flavonoid and proanthocyanidin contents of the plant were 0.618 and 0.004 mg gallic acid equivalent/g of extract powder, respectively, with reference to a standard curve (Y = 0.0067x + 0.0132, r^2^ = 0.999).

**Figure 1.**
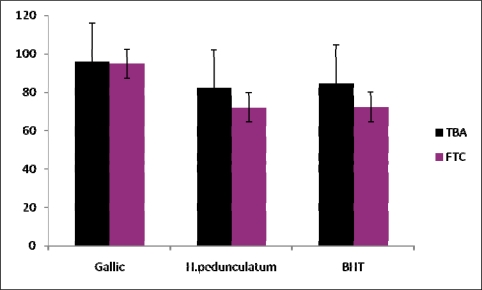
Antioxidant properties of extract compared to the standards (gallic acid and BHT) as determined with the FTC (500 nm) and TBA (552 nm) methods on the 6th day.

The *in vitro* antioxidant assay of the plant extract (Figure 1) reveals appreciable antioxidant potential compared with the standards BHT and gallic acid. The inhibition of lipid peroxide at the initial stage of oxidation was 82.14%, compared to BHT (84.6%) and gallic acid (96%), and the inhibition of malondialdehyde by the extract showed inhibition of 72% compared to both BHT (72.24%) and gallic acid (94.82%). Table 2 shows the reducing power of the aqueous extract in comparison with a BHT standard at 700 nm. The reducing capacity of the extract, another considerable indicator of antioxidant activity was also found to be substantial. The inhibition of scavenging activities of the aqueous extract for DPPH, ABTS, hydrogen peroxide, nitric oxide and superoxide anion radical are shown in Table 3. The ABTS and nitric oxide radical scavenging activity of the extract at 0.8 mg/mL (the highest concentration of the extract tested) was 77.8 and 68%. The extract showed appreciable free radical scavenging activities at the highest concentrations of 0.8 mg/mL on hydrogen peroxide, superoxide anion radical and DPPH with percentage inhibitions of 77.13%, 79% and 69.3% respectively (Table 3). All activities followed a concentration dependent manner and compared favourably well with the standard (BHT) at all concentrations.

### 2.2. Discussion

The analysis of aqueous extracts of the leaves of *H. pedunculatum* indicated the presence of phenolics, glycosides, flavonoids, proanthocyanidins and tannins. Phenol and phenolic compound such as flavonoids have been shown to possess significant antioxidant activities [28]. These compounds are known to be biologically active through different mechanisms; tannins for example, act by iron sequestration, hydrogen bounding or specific interactions with vital proteins such as enzymes [29]. Herbs containing tannins are astringent and used for treating intestinal disorders such as diarrhoea and dysentery [30]. The presence of tannins in *H. pedunculatum* supports the traditional medicinal use of this plant in the treatment of different diseases. Morta *et al*. [31] revealed the importance of tannins for the treatment of inflamed or ulcerated tissues. Kapil *et al*. [32] reviewed the biological activities of tannins and observed that tannins (whether total or pure compound) have remarkable activity in cancer prevention and anticancer activities. In addition to its antimicrobial, anticancer activities, tannins are potent antioxidants [14]. The observations above support the use of *H. pedunculatum* in herbal cure remedies. Steroids, abundant in many plants, have been shown to have hypercholesterolemic effects [33] and are used as emollients, diuretics and as a central nervous system depressant. They also exhibit anti-leukemic, antipyretic, anti-fungal, hypnotic, and muscle relaxant activities. Furthermore, the ribose derivatives of steroids are active as anticancer and anti-viral agents [33–35]. Steroids have been reported to stimulate menstrual discharge and diminish secretion of milk [33]. Flavonoids which are also among the constituents of *H. pedunculatum* leaves extract exhibit a wide range of biological activities which include antimicrobial, anti-inflammatory, anti-angionic, analgesic, anti-allergic effects, cytostatic and antioxidant properties [36]. Flavonoids’ ability of scavenging hydroxyl radicals, superoxide anion radicals and lipid peroxyradicals highlights many of their health-promoting functions in organism, which is important for prevention of diseases associated with oxidative damage of membranes, proteins and DNA [37]. Flavonoids in the human diet may reduce the risk of various cancers, as well as prevent menopausal symptoms [36]. Epidemiological studies suggest that the consumption of flavonoids is effective in lowering the risk of coronary heart diseases [38], thus, *H. pedunculatum* could be useful in treating coronary heart disease. Lastly, saponins which are responsible for numerous pharmacological properties [39] were also present in *H. pedunculatum* leaf extract. Saponins constitute a key ingredient in traditional Chinese medicine and are responsible for many of the attributed biological effects [40]. Saponins are known to produce inhibitory effect on inflammation [41]. Therefore, therapeutic effects of some medicinal plants commonly used in folklore remedies can be attributed to the antioxidant properties of their constituents. This is further corroborated by the result of our FTC and TBA antioxidant assays. Interestingly, the reduction in peroxide level at the concentrations investigated may indicate the ability of the herb to minimize oxidative damage to some vital tissues in the body [42,43].

In the reducing power assay, the presence of antioxidants in the sample result in the reduction of Fe^3+^ to Fe^2+^ by donating an electron. The amount of Fe^2+^ can then be monitored by measuring the formation of Perl’s blue at 700 nm. Increasing absorbance indicates an increase in reductive ability. The results show that there was increase in reducing power of the extract as the extract concentration increases.

Plants with antioxidant activities have been reported to possess free radical scavenging activity [44]. Free radicals are known as a major contributor to several clinical disorders such as diabetes mellitus, cancer, liver diseases, renal failure and degenerative diseases as a result of deficient natural antioxidant defense mechanism [2].

The result of DPPH scavenging activity assay in this study indicates that the extract was potently active. The ability of extract to scavenge DPPH could also reflect its ability to inhibit the formation of ABTS+. The scavenging activity of ABTS+ radical by the extract was found to be significant. The results obtained in this study were contrary to findings of a previous study [45] which reported that compounds which exhibit ABTS+ scavenging activity may not possess DPPH scavenging activity. This implies that the plant extract may be useful for treating radical-related pathological damage especially at higher concentration.

Superoxide anion radical is one of the strongest reactive oxygen species among the free radicals that could be generated; it also has the ability to change to other harmful reactive oxygen species and free radicals within the living cells [26]. The scavenging activity of this radical by the extract compared with the standard suggests that the plant is also a potent scavenger of superoxide radical.

Hydrogen peroxide is a highly important reactive oxygen species because of its ability to penetrate biological membranes. However, it may be toxic if converted to hydroxyl radical in the cell [46]. The extract was capable of scavenging hydrogen peroxide in a concentration dependent manner.

Nitric oxide (NO) is a reactive free radical produced by phagocytes and endothelial cells, to yield more reactive species such as peroxynitrite which can be decomposed to form OH radical. The level of nitric oxide was significantly reduced in this study by the extract. Since NO plays a crucial role in the pathogenesis of inflammation [47], this may explicate the use of *H. pedunculatum* for the treatment of inflammation and for wound healing. Other investigators [7,48–50] have reported similar phytochemicals and their antioxidant activities found in the *Helichrysum* genus, these corroborate our results from this experiment.

## 3. Experimental

### 3.1. Plant material

Leaves of *H. pedunculatum* were collected from the vicinity of the Research Farm of the University of Fort Hare, Alice, Eastern Cape Province of South Africa, during September 2007. A specimen was deposited at the Giffen’s Herbarium of the Plant Science building of the University of Fort Hare in Alice. The identity was confirmed by the curator of the Herbarium to be *H. pedunculatum.* The leaves were picked and washed with water, air-dried (30 °C), pulverized (Christy Lab Mill, Christy and Norris Ltd; Process Engineers, Chelmsford, England) and stored in a sterile air-tight container for further use.

### 3.2. Preparation of extract

The powdered plant material (200 g) was extracted in sterile distilled water (5.5 L) on shaker (Stuart Scientific Orbital Shaker, UK) for 48 hours. The extract was filtered using a Buchner funnel and Whatman No.1 filter paper. The filtrate was quickly frozen at −40 °C and dried for 48 h using a freeze dryer (Savant Refrigerated vapor Trap, RV T41404, USA) to give a yield of 30 g of dry extract. The resulting extract was reconstituted with sterile distilled water to give concentrations used in this study.

### 3.3. Phytochemical screening of the plant extract

A small portion of the dry extract was used for the phytochemical tests for compounds which include tannins, flavonoids, alkaloids, saponins, and steroids in accordance with the methods of [14,15] with little modifications.

### 3.4. Determination of total phenolic composition

The amount of phenolic compound in the aqueous leaf extract of *H. pedunculatum* was determined with Folin Ciocalteu reagent using the method of [16], modified by [17]. To 0.5 mL of each sample (three replicates) of plant extract solution (1 mg/mL) was added 2.5 mL of 10% Folin-Ciocalteu reagent and 2 mL of Na_2_CO_3_ (2% w/v). The resulting mixture was incubated at 45 °C with shaking for 15 min. The absorbance of the samples was measured at 765 nm using UV/visible light. Results were expressed as milligrams of gallic acid (0–0.5 mg/mL) dissolved in distilled water.

### 3.5. Estimation of total flavonoids

The aluminum chloride colorimetric method was used for flavonoid determination. One milliliter (1 mL) of sample was mixed with 3 mL of methanol, 0.2 mL of 10% aluminum chloride, 0.2 mL of 1 M potassium acetate and 5.6 mL of distilled water and remains at room temperature for 30 min. The absorbance of the reaction mixture was measured at 420 nm with UV visible spectrophotometer. The content was determined from extrapolation of calibration curve prepared with gallic acid solution (0–0.8 mg/mL) in distilled water. The concentration of flavonoid was expressed in terms of mg gallic acid equivalents/mL.

### 3.6. Determination of total proanthocyanidins

Total proanthocyanidins were determined based on the procedure of Sun *et al*. [18]. A mixture of 3 mL of vanillin-methanol (4% v/v) and 1.5 mL of hydrochloric acid was added to 0.5 mL (1 mg/mL) of aqueous extract and vortexed. The resulting mixture was allowed to stand for 15 min at room temperature followed by the measurement of the absorbance at 500 nm. Total proanthocyanidin content was expressed as gallic acid equivalent (mg/mL) from the standard curve.

### 3.7. Determination of reducing power

The reducing power of the extract was evaluated according to the method of Oyaizu [19]. The mixture containing 2.5 mL of 0.2M phosphate buffer (pH 6.6) and 2.5 mL of K_3_Fe(CN)_6_ (1% w/v) was added to 1.0 mL of the extract dissolved in distilled water. The resulting mixture was incubated at 50 °C for 20 min, following by the addition of 2.5 mL of trichloroacetic acid (TCA, 10% w/v). The mixture was centrifuged at 3000 rpm for 10 min to collect the upper layer of the solution (2.5 mL), mixed with distilled water (2.5 mL) and 0.5 mL of FeCl_3_ (0.1%, w/v). The absorbance was measured at 700 nm against blank sample.

### 3.8. Antioxidant assay

The antioxidant activity of the aqueous extract was determined using ferric thiocyanate (FTC) and thiobarbituric acid (TBA) methods [20,21]. The FTC method was used to measure the amount of peroxide at the beginning of peroxidation while TBA method was used to measure free radicals present after peroxide oxidation.

### 3.9. Ferric thiocyanate (FTC) method

The standard method described by Kikuzaki *et al*. [20] was used for FTC determination. The absorbance of the resulting mixture (red colour) was measured at 500 nm every 24 h until the absorbance of the control reached its maximum. Butylated hydroxyl toluene (BHT) was used as positive control. While the mixture without the extract was used as the negative control.

### 3.10. Thiobarbituric acid (TBA) method

The method of Ottolenghi [21] modified by Kikuzaki *et al*. [22] was used for the determination of free radicals present after peroxide oxidation of aqueous leaf extract. The final sample concentration of 0.02% w/v from the samples prepared for FTC assay was used. Two milliliters of 20% trichloroacetic acid and 2 mL of 0.67% of thiobarbituric acid were added to 1 mL of sample solution followed the FTC method. The mixture was placed in a boiling water bath for 10 min and then centrifuged after cooling at 3,000 rpm for 20 min. The absorbance activity of the supernatant was measured at 552 nm and recorded after the reaction has stopped.

### 3.11. 2,2-Diphenyl-1-picrylhydrazyl (DPPH) assay

The method of Liyana-Pathiana and Shahidi [23] was used for the determination of scavenging activity of DPPH free radical. To 1 mL of 0.135 mM DPPH prepared in methanol was mixed with 1.0 mL of aqueous extract ranging from 0.2–0.8 mg/mL. The reaction mixture was vortexed thoroughly and left in dark at room temperature for 30 min. The absorbance was measured spectrophotometrically at 517 nm. The scavenging ability of the extract was calculated using the standard equation [23].

### 3.12. 2,2’-Azino-bis(3-ethylbenzthiazoline-6-sulphonic acid (ABTS) scavenging activity

The method of Re *et al*. [24] was adopted for the determination of ABTS activity of the plant extract. The working solution was prepared by mixing two stock solutions of 7 mM ABTS solution and 2.4 mM potassium persulphate solution in equal amount and allowed to react for 12 h at room temperature in the dark. The resulting solution was later diluted by mixing 1mL of freshly prepared ABTS^.+^ solution followed by the measurement of absorbance at 734 nm after 7 min. The scavenging capacity of the extrac for ABTS^.+^ was calculated and compared with butylated hydroxyltoluene (BHT).

### 3.13. Scavenging activity of nitric oxide (NO)

The method of Garrat [25] was adopted to determine the nitric oxide radical scavenging activity of aqueous extract of *H. pedunculatum*. Sodium nitroprusside in aqueous solution at physiological pH spontaneously generate nitric oxide which interacts with oxygen to produce nitrite ions determined by the use of Griess reagents. To 2 mL of 10 mM sodium nitroprusside dissolved in 0.5 mL phosphate buffer saline (pH 7.4) was mixed with 0.5 mL of plant extract at various concentrations (0.2–0.8 mg/mL). The mixture was incubated at 25 °C. After 150 min, 0.5 mL of incubation solution was withdrawn and mixed with 0.5 mL of Griess reagent [(1.0 mL sulfanilic acid reagent (0.33% in 20% glacial acetic acid at room temperature for 5 min with 1 mL of naphthylethylenediamine dichloride (0.1% w/v)]. The mixture was incubated at room temperature for 30 min. The absorbance was measured at 540 nm. The amount of nitric oxide radical was calculated following this equation:

$$\%\;\text{inhibition}\;\text{of}\;\text{NO}\;=\;[{\text{A}}_{0}-{\text{A}}_{1}]/{\text{A}}_{0}\;\times \;100$$

where A_0_ is the absorbance before reaction and A_1_ is the absorbance after reaction has taken place.

### 3.14. Scavenging activity of superoxide anion

The scavenging activity of superoxide anion was determined by the method of Yen and Chen [26]. The reaction mixture of 1 mL of extract (1 mg/mL), 1 mL of PMS (60 μM) prepared in phosphate buffer (0.1 M pH 7.4) and 1 mL of NADH (phosphate buffer) was incubated at 25 °C for 5 min, the absorbance was read at 560 nm against blank samples.

### 3.15. Hydrogen peroxide scavenging activity

Scavenging activity of hydrogen peroxide by the extract was determined by the method of Ruch *et al*. [27]. Extract (4 mL) prepared in distilled water at various concentration was mixed with 0.6 mL of 4 mM H_2_O_2_ solution prepared in phosphate buffer (0.1M pH 7.4) and incubated for 10 min. The absorbance of the solution was taken at 230 nm against blank solution containing the extract without H_2_O_2._

## 4. Conclusions

In conclusion, etiological factors of several clinical disorders could be traced to a deficient natural antioxidant defense in an individual. These disorders can be prevented or delayed by supplementing the body’s natural antioxidant defense. Plant extracts and plant-derived antioxidant compounds potentiate body’s antioxidant defense, they are antioxidants of choice because of their lower toxicity and side effects over the synthetic ones. Also, they are relatively cheaper and are easily accessible.

Aqueous leaf extract of *H. pedunculatum* have shown *in vitro* antioxidant activities which may be due to the presence of flavonoids, phenolics and proanthocyanidins. A further study to characterize the active principles and to elucidate the mechanism of action of this extract is the subject of ongoing investigation in our group.

## Figures and Tables

**Table 1.** Components of *H. pedunculatum* based on the preliminary aqueous leaf extract screening.

**Compounds**	**Presence**	**Extract equivalent of Gallic Acid (mg/g)**
Tannins	+	ND
Flavonoids	++	ND
Steroids	+++	ND
Alkaloids	−	ND
Saponins	+	ND
Total phenol	+++	0.512
Total flavonoids	+++	0.618
Total proanthocyanidin	+	0.004

+++ = appreciable amount;

++ = moderate amount;

+ = trace amount;

− = completely absent; ND = not determined.

**Table 2.** Reducing power activities of the aqueous extract of *H. pedunculatum* in comparison with a standard (BHT) at λ = 700 nm.

**Absorbance (700 nm)**		
**Concentrations (mg/mL)**	**Plant extract**	**BHT**
0.2	0.100 ± 0.00	0.110 ± 0.10
0.4	0.330 ± 0.00	0.510 ± 0.00
0.6	0.471 ± 0.00	0.790 ± 0.00
0.8	0.560 ± 0.00	1.050 ± 0.00

**Table 3.** Radical scavenging activities of aqueous leaf extract of *H. pedunculatum* and BHT as standard at different concentrations.

**Percentage inhibition (% I) of radical scavenging of *H. pedunculatum***					
**Extract or BHT concentration (mg/mL)**	**Superoxide anion**	**Nitric oxide**	**DPPH**	**Hydrogen peroxide**	**ABTS**
0.2	52.50(60.16)	20.02(40.27)	36.00(42.62)	62.12(68.61)	45.19(51.17)
0.4	70.69(73.49)	38.49(46.27)	47.20(53.00)	67.16(73.29)	62.11(63.39)
0.6	74.70(77.12)	55.17(61.87)	64.60(73.99)	69.54(76.22)	75.33(77.20)
0.8	79.00(79.96)	68.00(80.29)	69.30(82.32)	77.13(80.00)	77.80(77.95)

BHT values in bracket.
